# Genomic biomarkers of prenatal intrauterine inflammation in umbilical cord tissue predict later life neurological outcomes

**DOI:** 10.1371/journal.pone.0176953

**Published:** 2017-05-11

**Authors:** Sloane K. Tilley, Robert M. Joseph, Karl C. K. Kuban, Olaf U. Dammann, T. Michael O’Shea, Rebecca C. Fry

**Affiliations:** 1 Department of Environmental Sciences and Engineering, Gillings School of Global Public Health, University of North Carolina, Chapel Hill, North Carolina, United States of America; 2 Department of Anatomy and Neurobiology, Boston University School of Medicine, Boston, Massachusetts, United States of America; 3 Department of Pediatrics, Boston University School of Medicine, Boston, Massachusetts, United States of America; 4 Department of Public Health and Community Medicine, Tufts University School of Medicine, Boston, Massachusetts, United States of America; 5 Department of Pediatrics, University of North Carolina, Chapel Hill, North Carolina, United States of America; 6 Curriculum in Toxicology, School of Medicine, University of North Carolina, Chapel Hill, North Carolina, United States of America; Hopital Robert Debre, FRANCE

## Abstract

**Background:**

Preterm birth is a major risk factor for neurodevelopmental delays and disorders. This study aimed to identify genomic biomarkers of intrauterine inflammation in umbilical cord tissue in preterm neonates that predict cognitive impairment at 10 years of age.

**Study design:**

Genome-wide messenger RNA (mRNA) levels from umbilical cord tissue were obtained from 43 neonates born before 28 weeks of gestation. Genes that were differentially expressed across four indicators of intrauterine inflammation were identified and their functions examined. Exact logistic regression was used to test whether expression levels in umbilical cord tissue predicted neurocognitive function at 10 years of age.

**Results:**

Placental indicators of inflammation were associated with changes in the mRNA expression of 445 genes in umbilical cord tissue. Transcripts with decreased expression showed significant enrichment for biological signaling processes related to neuronal development and growth. The altered expression of six genes was found to predict neurocognitive impairment when children were 10 years old These genes include two that encode for proteins involved in neuronal development.

**Conclusion:**

Prenatal intrauterine inflammation is associated with altered gene expression in umbilical cord tissue. A set of six of the differentially expressed genes predict cognitive impairment later in life, suggesting that the fetal environment is associated with significant adverse effects on neurodevelopment that persist into later childhood.

## Introduction

Preterm birth, defined as delivery at < 37 completed weeks gestation, is currently the leading cause of neonatal morbidity and mortality in the United States [1]. Individuals born prematurely are at increased risk for other adverse health outcomes, and those born at less than 28 weeks gestation are at particularly high risk [2]. Perhaps most important are adverse neurodevelopmental outcomes, which affect an estimated 1 million preterm infants born each year [3].

Preterm birth is thought to be caused by the pathological induction of certain components of the normal parturition process resulting from a combination of environmental, genetic, and behavioral factors [4,5]. Many identified risk factors have the potential to promote inflammatory processes [6]. Indicators of intrauterine inflammation are present in as many as 40–70% of preterm births, versus only 1–13% of full term births [7]. These data support the hypothesis that risk of preterm birth is increased by pathological, environmental, and/or genetic factors that contribute to delivery-inducing inflammation [4,8]. Among preterm infants, biomarkers of prenatal inflammation, including inflammatory cytokines in amniotic fluid [9], placental histologic findings [10–12], and inflammation-related proteins in neonatal blood [13–17], are associated with a range of neurodevelopmental impairments [18].

A fetal inflammatory response (FIR) is associated with increased expression of a broad array of genes related to neurodevelopment [19]. In the present study, we aimed to identify whether genomic signaling changes in umbilical cord tissue were associated with a suite of four histologic markers of prenatal inflammation in a subset of infants from the Extremely Low Gestational Age Newborns (ELGAN) cohort. We hypothesized that some of these genomic changes would be predictive of neurocognitive function at 10 years of age and could provide novel predictive biomarkers of neurocognitive impairment in preterm infants.

## Materials and methods

### The ELGAN cohort

The ELGAN cohort was established to identify risk factors for neurodevelopmental impairments in extremely low gestational age newborns. Between 2002–2004, 1506 infants were enrolled in the study, and in 1410 cases (94% of the cohort) placentas were collected for pathological examination. Placentas were collected at delivery and flash frozen to -70 C. Placentas were examined both grossly and histologically for many parameters, including a subset of intrauterine inflammation markers. The larger ELGAN cohort is described in detail elsewhere [20,21].

### RNA isolation and gene expression assessment

The data in this study were generated by Cohen *et al*. from isolated total RNA from the umbilical cord tissue homogenates collected from infants born between 23 and 28 weeks gestation at Brigham and Women's Hospital, Beth Israel Deaconess Medical Center, or Wake-Forest Medical Center between April 1, 2004 and August 31, 2004. RNA was extracted using the Qiagen RNeasy Mini Kit, as described elsewhere [22]. Thirteen samples had insufficient total RNA (< 7 μg) for hybridization and five infants died before 36 weeks postmenstrual age. These 18 infants were excluded from further analysis. Total RNA from the 54 remaining ELGAN subjects were hybridized to the Affymetrix Human Genome U133 Plus 2.0 Array, which assesses gene expression levels across 54,675 probes. All expression data are available at the National Center for Biotechnology Information's Gene Expression Omnibus repository (GSE8586) [22]. The data were further processed previously, applying quality control assessments to all 54 samples [19]. Six samples that failed these measures were excluded from further analysis [19]. Data were normalized into Affymetrix probesets using fRMA [19]. An additional five subjects were excluded from analysis due to missing clinical information about intrauterine inflammation markers from the umbilical cord tissue. Probes without annotations to *Entrez* gene identifiers were removed and only the probeset with the largest inter quartile range per *Entrez* gene was kept. The final data set consisted of measures of gene expression across 20,155 genes for n = 43 subjects [19] (S1 Table).

### Cognitive assessment at 10 years of age

When study participants were 10 years of age, general cognitive ability (or IQ) was assessed with the School-Age Differential Ability Scales–II (DAS-II) Verbal and Nonverbal Reasoning scales [23]. Attention and executive function were assessed with the DAS-II and the NEPSY-II [24]. DAS-II Recall of Digits Backward and Recall of Sequential Order measured verbal working memory. NEPSY-II Auditory Attention and Auditory Response Set evaluated auditory attention, set switching and inhibition. NEPSY-II Inhibition and Inhibition Switching assessed simple inhibition and inhibition in the context of set shifting, respectively. NEPSY-II Animal Sorting measured concept generation and mental flexibility. As a comprehensive measure of cognitive and executive function, we used latent profile analysis (LPA) to identify study participants with similar distinctive profiles on measures of cognitive and executive functioning. With this approach, four subgroups were identified, corresponding to functioning that was normal (LPA score = 1; 34% of ELGAN cohort), low-normal (LPA score = 2; 41%), moderately impaired (LPA score = 3; 17%), and severely impaired (LPA score = 4; 8%) [25,26].

### Relating gene expression to prenatal inflammation measures

Four markers of intrauterine inflammation were selected to test for associations with gene expression: inflammation of the chorionic plate, moderate or severe chorioamnionitis, neutrophilic infiltration of the fetal vessels in the chorionic plate, umbilical cord inflammation [27]. Using ANCOVA analysis, these binary measures were assessed separately. Potential confounders were included in the ANCOVA analysis for each placental histologic marker only if they displayed different means (2-sided student t-test p-value < 0.20) between subjects with that placental inflammation marker and without that placental marker. Variables tested but that displayed no mean difference in any of the four histologic markers of placenta inflammation included maternal age, maternal race, maternal BMI, maternal education level and infant sex. Exposure to smoke (active or passive) during pregnancy was included in the analysis for chorioamnionitis and neutrophilic infiltration of the fetal vessels, and gestational age was also included in the analysis for neutrophilic infiltration of the fetal vessels. In order to control for multiple tests, false discovery rate (FDR) q-values were calculated. Significance was defined as FDR q-value < 0.05 and an absolute fold change ≥ |2.0|.

### Network analysis of genes associated with prenatal inflammation measures

In order to examine the higher-level biological functions and processes related to genes changed in association with four markers of prenatal intrauterine inflammation, functional relationships were assessed among these genes using Ingenuity Network Analysis (IPA) (Ingenuity Systems^®^, Redwood City, CA, USA) and STRING v10.0 [28,29]. Network analyses were stratified by directionality of gene expression associations. Canonical pathways from IPA and PFAM protein domains enriched among these gene sets were analyzed and reported.

### Logistic regression of genomic markers of inflammation to later life neurological score

We tested whether the expression levels of genes associated with one or more intrauterine inflammation marker predicted later life neurocognitive function using exact logistic regression analysis. Exact logistic regression was used due to the small subset of subjects for whom LPA score measured (n = 22). The dependent variable was the child’s LPA score at age 10, with expression levels predicting the binary outcome of (i) no or low impairment (LPA score = 1 or 2, n = 17) or (ii) moderate or severe impairment (LPA score = 3 or 4, n = 5). As potential confounders had been controlled for in the first step of this analysis, the model was run with gene expression as the sole predictor variable. Significance was defined as an exact p-value < 0.05, and exact beta estimates, exact parameter-likelihood odds ratios and 95% confidence intervals for odds ratios are reported.

## Results

### Characteristics of the study cohort

Maternal and infant demographic and birth clinical data are presented in Table 1 for the subset of the ELGAN subjects with gene expression data used in this study (n = 43). In these infants, the majority of mothers were white and reported no smoking during pregnancy. The mean week of delivery was 26.1, and approximately two-thirds of the infants were male (Table 1). The demographic characteristics in this subset of ELGAN infants were similar to those reported for all ELGAN subjects [21], with the exception of a higher proportion of males in this cohort. We calculated the prevalence of four histological markers of prenatal intrauterine inflammation: inflammation of the chorionic plate, neutrophilic infiltration of the fetal vessels in the chorionic plate, and umbilical cord inflammation were all present in approximately 25% of the study subjects, while moderate or severe chorioamnionitis was present in approximately 50% of the study subjects (Table 1). The occurrence of these four histologic markers of inflammation were consistent with those reported in larger study (n = 947) of the ELGAN cohort [20].

**Table 1:** Maternal and child characteristics. Data are number of study participants (percent) except where indicated.

Characteristic	N = 1410 ELGAN Subjects	N = 43 ELGAN Subjects	N = 22 ELGAN Subjects
Maternal Age at Delivery in years (median; range in parenthesis)	28.6 (13.2–47.3)	32.1 (15.8–43.2)	34 (19.4–43.2)
Maternal Race			
White	819 (58.1%)	26 (60.5%)	18 (81.8%)
African-American	397 (28.2%)	9 (20.9%)	4 (18.2%)
Other	178 (12.6%)	7 (16.3%)	0 (0%)
Unknown	16 (1.1%)	1 (2.3%)	0 (0%)
Pre-pregnancy BMI (kg/m^2^) (median; range in parenthesis)	23.9 (13.2–72.1)	23.1 (18.1–46.5)	22.5 (19.1–46.5)
Public Health Insurance			
No	785 (55.7%)	31 (72.1%)	19 (86.3%)
Yes	558 (39.6%)	12 (27.9%)	3 (13.6%)
Unknown	67 (4.8%)		
Education			
< = 12 years	224 (15.9)	10 (23.3%)	3 (13.6%)
12–15 years	681 (48.3%)	12 (27.9%)	7 (31.8%)
16+ years	404 (28.7%)	20 (46.5%)	11 (50.0%)
Unknown	101 (7.2%)	1 (2.3%)	1 (4.5%)
Infertility Treatment			
No	1063 (75.4%)	31 (72.1%)	14 (63.6%)
Yes	264 (18.7%)	12 (27.9%)	8 (36.4%)
Unknown	83 (5.9%)		
Smoking during Pregnancy			
No	1133 (80.4%)	40 (93.0%)	20 (90.9%)
Yes	199 (14.1%)	3 (7.0%)	2 (9.1%)
Unknown	78 (5.5%)		
Infant Sex			
Male	752 (46.7%)	27 (62.8%)	14 (63.6%)
Female	658 (46.7%)	16 (37.2%)	6 (27.3%)
Birth and Later Life Outcomes			
Gestational Age (weeks)			
Median (range)	28.6 (13.2–47.3)	27 (23–27)	27 (23–27)
23–24 weeks	387 (27.5%)	6 (14.0%)	2 (9.1%)
25–26 weeks	618 (43.8%)	14 (32.6%)	8 (36.4%)
27 weeks	405 (28.7%)	23 (53.5%)	12 (54.5%)
Birth weight (g) (median; range in parenthesis)	790 (280–1528)	929.7, 952 (550–1360)	889.5 (550–1360)
Inflammation of the chorionic plate (Stage: 3 and Severity: 3)			
No	1118 (79.3%)	32 (74.4%)	16 (72.7%)
Yes	265 (18.8)	11 (25.6%)	6 (27.3%)
Unknown	27 (1.9%)		
Moderate/Severe Chorioamnionitis			
No	879 (62.3%)	23 (53.5%)	13 (59.1%)
Yes	505 (35.8%)	20 (46.5%)	9 (40.9%)
Unknown	26 (1.8%)		
Neutrophilic infiltration of fetal vessels in the chorionic plate			
No	1034 (73.3%)	29 (67.4%)	15 (68.2%)
Yes	340 (24.1%)	14 (32.6%)	7 (31.8%)
Unknown	36 (2.6%)		
Umbilical cord inflammation (grade 3–5)			
No	1136 (80.1%)	31 (72.1%)	16 (72.7%)
Yes	216 (15.3%)	12 (27.9%)	6 (27.3%)
Unknown	58 (4.1%)		
LPA Score			
1	282 (20.0%)	13 (30.2%)	13 (59.1%)
2	337 (23.9%)	4 (9.3%)	4 (18.2%)
3	134 (9.5%)	4 (9.3%)	4 (18.2%)
4	66 (4.7)	1 (2.3%)	1 (4.5%)
Unknown	591 (41.9%)	21 (48.8%)	0 (0%)

Maternal demographic data, pregnancy characteristics, and data on birth and later in life outcomes are presented for the entire ELGAN sample for which placentas were collected (N = 1410), as well as the N = 43 and N = 22 ELGAN subjects used in this analysis. Data are presented as the number (%) of subjects in the cohort unless otherwise noted. For each of the four histological markers of intrauterine inflammation, there was no significant difference in maternal age, maternal race, maternal BMI, maternal education level and infant sex between subjects with and without each marker (Student’s 2-sided t-test p-value > 0.20).

### Markers of prenatal inflammation are associated with umbilical cord gene expression

**Fig 1:**
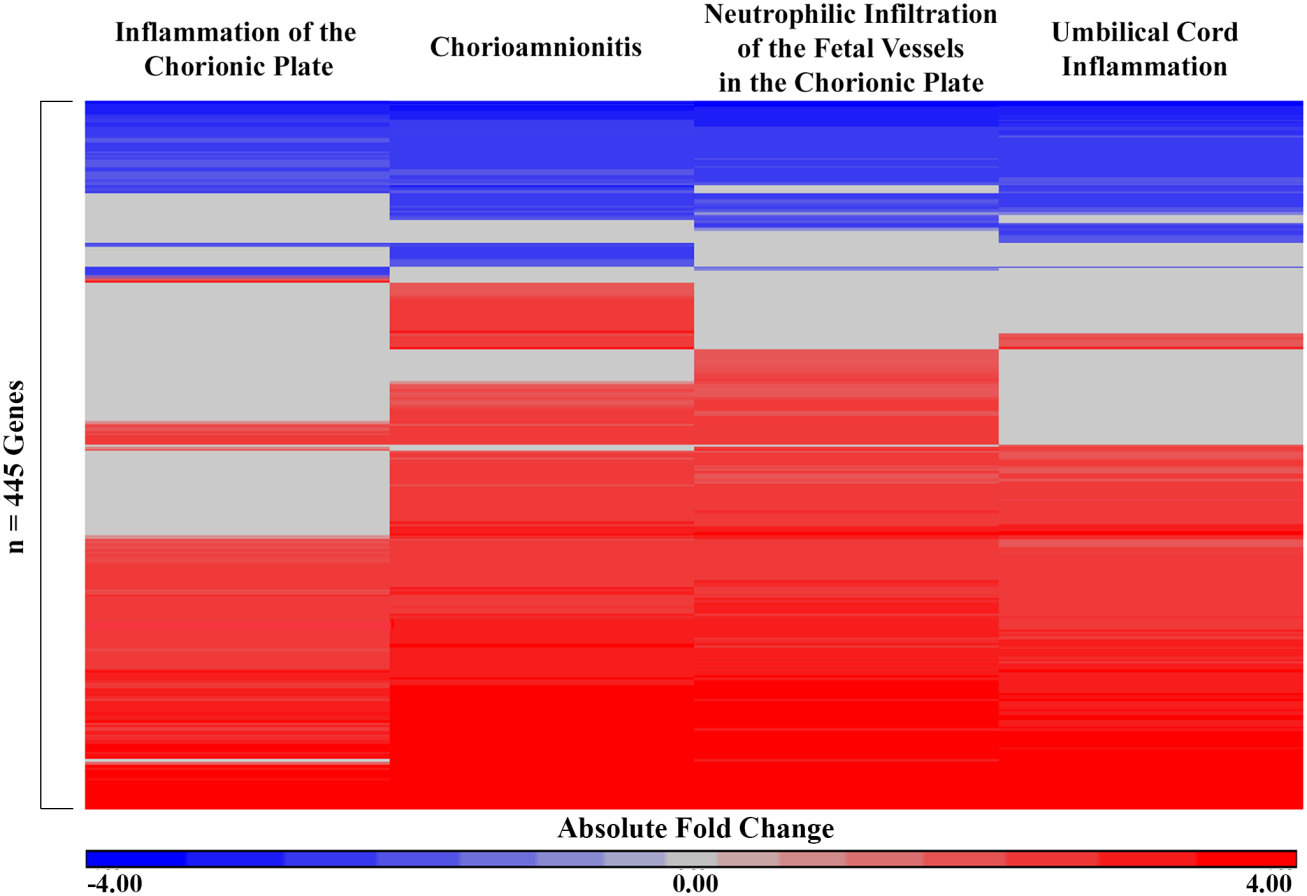
Heatmap of 445 genes that displayed significant differential expression values across one or more markers of intrauterine inflammation in n = 43 subjects. The absolute fold change (cases/controls) is displayed for each gene significantly associated with each marker of intrauterine inflammation. Red indicates increased expression in association with a marker of intrauterine inflammation and blue indicates decreased expression in association with a marker of intrauterine inflammation. Rows (genes) were organized by unsupervised hierarchical clustering Euclidean dissimilarity with average linkage. Significance was defined as FDR q-value < 0.05 and fold change ≥ |2.0|.

A comparison of mRNA expression between subjects with or without each of the four markers of intrauterine inflammation identified 445 unique genes (S1 Table). Among these 445 genes, 334 (75.1%) were increased in expression in association with at least one histological marker of intrauterine inflammation, and 111 (24.9%) were decreased in expression in association with at least one histological marker of intrauterine inflammation (Fig 1). All genes demonstrated remarkable consistency in the directionality of expression when associated with more than one marker of intrauterine inflammation (Fig 1). Inflammation of the chorionic plate was associated with the expression levels of 255 genes; moderate or severe chorioamnionitis was associated with the expression levels of 396 genes; neutrophilic infiltration of the fetal vessels in the chorionic plate was associated with the expression levels of 365 genes; and umbilical cord inflammation was associated with the expression levels of 323 genes. In addition, 221 genes were associated with the same directionality across all four intrauterine inflammation markers. Specifically, 168 of these 221 genes (76.0%) were increased in expression in association with the four intrauterine inflammation markers, 53 of these 221 (24.0%) genes were decreased in expression (S1 Table).

### Umbilical cord tissue gene expression profiles are enriched for inflammation and neuronal development processes

Systems level analysis was used to characterize the canonical pathways and protein domains enriched within the 334 genes with increased expression in association with one or more histological marker of intrauterine inflammation and the 111 genes with decreased expression in association with one or more histological marker of intrauterine inflammation. Notably, differences in functional signaling between the genes with increased and decreased expression were observed (Table 2). Specifically, the most significant canonical pathway enriched among the genes with increased expression was granulocyte adhesion and diapedesis, and the other top pathways were also associated with inflammation signaling (Table 2). In contrast, the most significant canonical pathways among the genes with decreased expression included gap junction signaling and dopamine-DARPP32 feedback in cAMP signaling, both of which are known to play important roles in early neuronal development (Table 2) [30–32]. Similarly, the protein domains that were significantly enriched among the genes with increased expression in association with one or more histological marker of intrauterine inflammation were small cytokines, metallothionein, and S-100/ ICaBP type calcium binding domain. Proteins containing all of these domains have been demonstrated to play a role in placental inflammation [33–35]. Interestingly, elevated levels of interleukin-8 (IL-8) in cord blood have been previously associated with a higher incidence with brain injury in preterm infants with placental inflammation [35]. Among genes with decreased expression, immunoglobulin I-set protein domains, which are known to function in nervous system development, were found to be significantly enriched [36]. Specifically, among the genes associated with a marker of intrauterine inflammation that encode for a protein with an immunoglobulin I-set protein domain and that have been previously indicated in neurodevelopment were contactin 1 (*CNTN1*), neuronal growth regulator 1 (*NEGR1*), neurotrophic receptor tyrosine kinase 3 (*NTRK3*), and receptor tyrosine kinase like orphan receptor 1 (*ROR1*) [37–40].

**Table 2:** Top canonical pathways and protein domains enriched among the 445 genes associated with intrauterine inflammation markers.

	IPA Canonical Pathways	p-value	Associated Genes	PFAM Protein Domains	p-value	Associated Genes
**Genes with Increased Expression Levels**	Granulocyte Adhesion and Diapedesis	1.58e-23	*IL1RL1*, *ITGAM*, *MMP9*, *SELL*, *CSF3R*, *CCL20*, *IL1R2*, *ITGB2*, *SELE*, *FPR1*, *VCAM1*, *CXCL6*, *CXCR4*, *IL1RN*, *IL1R1*, *C5AR1*, *CXCL1*, *CXCL5*, *CCL5*, *IL1B*, *TNFRSF1B*, *CXCL2*, *PPBP*, *CXCL8*, *TNFRSF11B*, *CCL2*, *SELPLG*, *MMP10*, *CXCL3*, *CSF3*, *ICAM1*	Small cytokines (intecrine/ chemokine), interleukin-8 like	1.13e-4	*CCL2*, *CCL20*, *CCL5*, *CXCL1*, *CXCL2*, *CXCL5*, *IL8*
	Hepatic Fibrosis / Hepatic Stellate Cell Activation	1.58e-16	*IL1RL1*, *MMP9*, *IL1R2*, *IL6*, *VCAM1*, *IL10RA*, *IFNGR1*, *IL1R1*, *FLT1*, *IGFBP3*, *CCL5*, *IL1B*, *VEGFA*, *PDGFRA*, *CD14*, *TNFRSF1B*, *CXCL8*, *NFKB2*, *TNFRSF11B*, *COL11A1*, *CCL2*, *CXCL3*, *TIMP1*, *SERPINE1*, *ICAM1*	Metallothionein	4.27e-2	*MT1H*, *MT1X*, *MT2A*
	Atherosclerosis Signaling	7.94e-16	*CCR2*, *S100A8*, *IL1B*, *MMP9*, *ITGB2*, *SELE*, *IL6*, *VCAM1*, *CXCL8*, *ALOX5*, *NFKB2*, *LYZ*, *CXCR4*, *IL1RN*, *CCL2*, *SELPLG*, *SERPINA1*, *F3*, *PLBD1*, *ICAM1*, *PLA2G2A*	S-100/ ICaBP type calcium binding domain	4.27e-2	*S100A4*, *S100A8*, *S100A9*, *S100P*
**Genes with Decreased Expression Levels**	Gap Junction Signaling	2.04e-5	*CAV1*, *GUCY1A2*, *GUCY1A3*, *GAB1*, *PLCE1*, *PRKAG2*, *PLCL1*	Immunoglobulin I-set domain	1.83e-3	*CNTN1*, *NEGR1*, *NTRK3*, *OPCML*, *ROR1*
	Cellular Effects of Sildenafil (Viagra)	4.57e-5	*MYH3*, *GUCY1A2*, *PLCE1*, *GUCY1A3*, *PRKAG2*, *PLCL1*			
	Dopamine-DARPP32 Feedback in cAMP Signaling	1.55e-4	*GUCY1A2*, *GUCY1A3*, *PPM1L*, *PLCE1*, *PRKAG2*, *PLCL1*			

The top three most significant pathways (right-tailed Fisher’s Exact test p-value < 0.0001) and significant protein domains (FDR p-value < 0.05) are listed. Network analyses were stratified by gene expression directionality. Genes that displayed increased expression levels in association with one or histological markers of intrauterine inflammation were enriched for canonical pathways and protein domains involved in inflammatory and immune processes. Genes that displayed decreased expression levels in association with one or histological markers of intrauterine inflammation were enriched for canonical pathways and protein domains involved early neuronal development.

### Genomic changes in umbilical cord tissue are related to neurocognitive function

**Fig 2:**
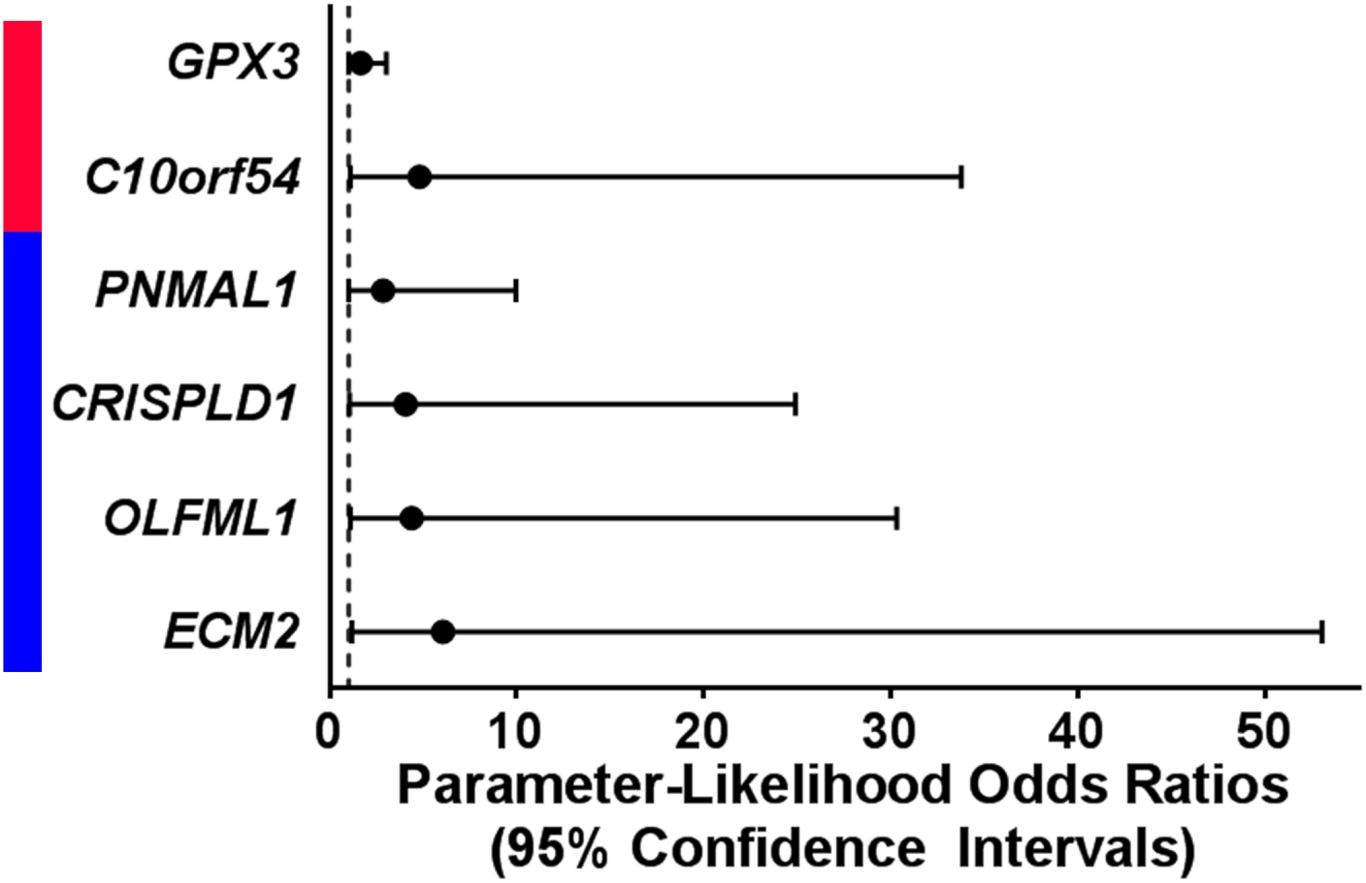
Expression levels of six genes predict neurocognitive outcome at 10 years of age. Increased levels (red) of two genes that were associated with a maker of intrauterine inflammation predicted more severe neurocognitive at 10 years of age. Decreased levels (blue) of 4 genes that were associated with a maker of intrauterine inflammation predicted more severe neurocognitive at 10 years of age. Significance was defined as an exact p-value < 0.05 in a logistic regression model.

In exact logistic regression models testing all 445 genes, expression levels of 6 genes altered in association with one or more histological marker of prenatal intrauterine inflammation also predicted neurocognitive impairment later in life (Fig 2, S2 Table). Increased expression levels of chromosome 10 open reading frame 54 (*C10orf54*, p = 3.64e-2) and glutathione peroxidase 3 (*GPX3*, p = 4.53e-2) predicted greater neurocognitive impairment later in life and were also increased in association with at least one marker of prenatal intrauterine inflammation (Fig 2). Decreased expression levels of cysteine rich secretory protein LCCL domain containing 1 (*CRISPLD1*, p = 4.06e-2), extracellular matrix protein 2 (*ECM2*, p = 2.86e-2), olfactomedin like 1 (*OLFML1*, p-value = 3.36e-2), and paraneoplastic Ma antigen family like 1 (*PNMAL1*, p-value = 4.89e-2) predicted more severe neurocognitive impairment and were also decreased in association with at least one marker of prenatal intrauterine inflammation (Fig 2). *CRISPLD1* and *ECM2* have been suggested to play roles in neuronal development [41,42].

## Discussion

In this study, we have demonstrated evidence of genomic signaling changes in the umbilical cord tissue of extremely preterm infants that are associated with multiple markers of intrauterine inflammation. Two interesting patterns of gene expression were observed; inflammation-associated genes displayed increased expression in the cord, while among the genes that displayed decreased expression, several were related to neurodevelopment. Expression levels of six genes altered in umbilical cord tissue in association with one or more intrauterine inflammation marker significantly predict the risk of neurocognitive impairment later in life. In support of our data, several of these genes whose decreased expression predicted more severe cognitive impairment have been previously implicated in neuronal development. Our results indicate that genomic changes observable at parturition in the umbilical cord tissue of extremely low gestational age newborns are associated with neurocognitive function later in life.

Preterm newborns are at increased risk for numerous adverse health effects, many of which are related to prenatal intrauterine inflammation, including neurodevelopmental impairment [43,44]. In our study, we identified that genes that play a role in fetal neurodevelopment had decreased expression levels in the cord of infants exposed to the prenatal inflammatory environment. Broadly, these genes are associated with gap junction signaling and dopamine-DARPP32 feedback in cAMP signaling, both of which are associated with neurodevelopment. Gap junctions signal for crucial processes in the neonatal cerebral cortex, including neuronal proliferation, migration, and differentiation, while dopamine signaling is known to influence neuronal migration and dendritic growth [30,31]. Through the secondary analysis of genomic prediction of cognitive function, a total of six genes were identified. Two of these genes that displayed decreased expression in relation to inflammation, namely *ECM2* and *CRISPLD1*, have been previously indicated in neurodevelopment processes. *ECM2* encodes an extracellular matrix protein of the small leucine rich glycoprotein family, which are involved in the regulation of many phases of embryonic neurodevelopment [42]. *CRISPLD1* has also been reported to play a role in extracellular matrix regulation, which is known to play crucial roles in axon growth and guidance [45]. Changes in the expression levels of genes known to play a role in neuronal development in association with markers of prenatal inflammation contribute to the growing body of literature that supports an association between intrauterine inflammation and cognitive impairment in later childhood [18,35].

A previous study of this ELGAN study subset extensively described the robust genomic response that is measurable in umbilical cord tissue in relation to FIR [19]. In that study, FIR was defined as the presence of neutrophils in both the umbilical cord and chorionic plate, and the authors reported associations between FIR and gene expression levels of many genes, including 434 of the 445 (97.5%) genes reported here [19]. In support of our findings, this previous analysis similarly found that genes with decreased expression in association with FIR were enriched for roles in neurodevelopment [19]. Providing new information on pathways that drive later life disease, our data indicate that decreased expression levels of genes related to an inflammatory intrauterine environment are associated with adverse neurocognitive development in children who are born prematurely.

A possible limitation of our study is our small sample size due to missing cognitive data for 21 subjects with available gene expression data. The percentage of subjects in our subset of ELGAN infants that were lost to follow-up (21/43 = 48.8%) was much higher than that of the larger ELGAN cohort (8%) [25]. However, previous studies suggest that loss-to-follow-up percentages below 60% typically are not associated with substantial bias [46]. In order to compensate for our sample size, we employed exact logistic regression models, as recommended in cases when data are sparse [47]. Despite the modest sample size, we were able to detect associations between umbilical cord genomic markers and neurocognitive function ten years after birth.

In summary, we identified genes whose expression levels are associated with both intrauterine inflammation and later-life neurocognitive impairment. Identification of genes that are associated with adverse neurodevelopment could allow for improved surveillance for neurocognitive deficits and earlier intervention for children who are at risk, which has been shown to be an effective treatment method for children with intellectual disabilities [48]. Future research studies could aim to identify the underlying mechanisms for the altered gene expression patterns in order to mitigate the risk for cognitive impairment in extremely low gestational age newborns exposed to intrauterine inflammation.

## Supporting information

S1 Table Processed microarray data and demographical characteristics for the n = 43 ELGAN subjects used in the present analysis. (TXT)

S2 Table Genes associated with markers of intrauterine inflammation. 445 genes displayed altered expression levels in association with at least one of the following markers of intrauterine inflammation: inflammation of the chorionic plate, moderate or severe chorioamnionitis, neutrophilic infiltration of the fetal vessels in the chorionic plate, umbilical cord inflammation. Significance was defined as an ANCOVA FDR q-value < 0.05 and an absolute fold change ≥ |2.0|.
(XLSX)

S3 Table Results from exact logistic regression analysis. Six genes predicted a significantly greater risk of neurocognitive impairment (LPA score = 3 or 4 versus LPA score = 1 or 2) in exact logistic regression models. Statistics for *GPX3* and *C10orf54* are presented for logistic regression models where increased levels of gene expression predicted more severe neurocognitive impairment at 10 years of age. Statistics for *PNMAL1*, *CRISPLD1*, *OLFML1* and *ECM2* are presented for logistic regression models where decreased levels of gene expression predicted more severe neurocognitive impairment at 10 years of age.
(XLSX)
